# Supplementary material for: Patterns of Intron Gain and Loss in Fungi
Source: PLoS Biol. 2004 Nov 30;2(12):e422. doi: 10.1371/journal.pbio.0020422 (PMC532390; doi:10.1371/journal.pbio.0020422)
Supplement: Table S1 — Also available at http://genes.mit.edu/NielsenEtAl/. (4.3 MB ZIP). [file pbio.0020422.st001.zip › NielsenEtAl/html/55.html]

AN5895.1.NCU05288.1.MG07137.1.FG06166.1


```
 CLUSTAL W (1.82) Multiple Sequence Alignments - Introns Inserted


Sequence 1: NCU05288.1	465 aa
Sequence 2: FG06166.1	464 aa
Sequence 3: MG07137.1	464 aa
Sequence 4: AN5895.1	468 aa
Alignment Length: 470 aa
Number Identitical Residues: 325 aa
Alignment Score (without introns) 14342


MG07137.1 	-MEEIAKEYDVIVLGTG1LTECILSG2VLSVKGKKVLHIDRNDHYGG2EAASLNLQA0LF
NCU05288.1	MADEIAKEYDVIVLGTG~LTECILSG~VLSVKGKKVLHIDRNDHYGG2EAASVNLET~LF
FG06166.1 	-MDEIAPEYDVVVLGTG1LTECILSG2VLSVKGKKVLHIDRNDHYGG2EAASVNLET0LF
AN5895.1  	-MDEIAPEYDVIVLGTG1LTECVLSG2VLSVKGNKVLHIDRNDHYGG2EAASVNIET0LF
          	  :*** ****:***** ****:*** ******:************* ****:*::: **

MG07137.1 	KKYGNYNSGEEPWAKYGRNTDWNIDLVPKFLMSSGELTNILVSTDVTRYLEFKQVAGSFV
NCU05288.1	KKYGNFAAGTEPWKEYGRPNDWNIDLVPKFLMSSGELTNILVSTDVTRYLEFKQVAGSYV
FG06166.1 	KKYGNFREGEEPWKQYGRLNDWNIDLVPKFLMSSGELTNILVSTDVTRYLEFKQVAGSYV
AN5895.1  	KKYGNVRPGEEPWKKYGRVNDWNVDLVPKLLMANGELTNILVSTDVTRYLEFKQIAGSYV
          	*****   * *** :*** .***:*****:**:.********************:***:*

MG07137.1 	QQGSGPKATVAKVPSDAAEALRSPLMGIFEKRRMKSFIEWVGTFDLKDPATHKG1LDLSS
NCU05288.1	QQGAGSKATIAKVPSDAAEALRSPLMGIFEKRRMKSFIEWVGEFDPKDPATHKG1LDMAT
FG06166.1 	QQGASNKATVAKVPSDAAEALRSPLMGLFEKRRMKSFIEWVGSFDPKDPSTHKG1LDMNK
AN5895.1  	QQGKGPKATVAKVPSDAGEALRSSLMGMFEKRRAKKFLEWVGEFKEDDPSTHQG1LNIAT
          	*** . ***:*******.*****.***:***** *.*:**** *. .**:**:* *:: .

MG07137.1 	CTMKDVYDKFGLETGTKDFIGHAMALYLSDDYISKPGGAPEAIDRIRLYGNSVARYGKSP
NCU05288.1	CTMKDVFEKFSLEAGTKDFVGHAMALYLNDNYLDTPGAAPETIERIRLYGQSVARYGKSP
FG06166.1 	CTMKDVYDKFGLEATTKDFIGHAMALYLTDDYITTPGQAPEAIDRIRLYGNSVARYGKSP
AN5895.1  	CTMKDVYDKFSLEDNTRDFVGHSMALYQDDDYINRPGTASDAINRIRLYVNSMARYGKSP
          	******::**.**  *:**:**:****  *:*:  ** *.::*:***** :*:*******

MG07137.1 	YIYPLFGLGDLPQGFARLSAIYGGTYMLNTNIDELLYE-DGKAVGIKATMTGIEP---EM
NCU05288.1	YIYPLYGLGELPQGFARLSAIYGGTYMLNTNVDELVYE-NGKAVGIKATMTGVEP---EM
FG06166.1 	YIYPLYGLGELPQGFARLSAIYGGTYMLNTNIDEIQYD-GDKAVGIKATMTGVE----EM
AN5895.1  	YIYPLYGLGELPQGFARLSAIYGGTYMLNTDIDEVLYDENGKVSGIKATMKDREDGGETM
          	*****:***:********************::**: *:...*. ******.. * ... *

MG07137.1 	KFETRAKMILGDPSYFSDKVKVVGHVLRAICVLKHPLAGTSDADSCQLIIPQSQVGRKND
NCU05288.1	KFETRAKMILGDPSYFPDKVKVVGHVLRAICILKHPLASTNDADSCQLIIPQSQVGRKND
FG06166.1 	KFETKAKMILGDPSYFPNKAKVVGHVLRAICILKHPLAGTNDADSAQLIIPQSQVGRKND
AN5895.1  	KFETKTKKIIADPSYFPGKVSVSGYLLKAICILKHPIDKTDGSDSLQLIIPQSQVGRKRD
          	****::* *:.*****..*..* *::*:***:****:  *..:** ************.*

MG07137.1 	1IYIACVSSAHSVCPKGYYIAIVSTIAETSANHHLELQPGLERLGKIEEQFM0GPPIPLY
NCU05288.1	1IYIACVSSAHNVCPKGYWIAIVSTIAETSANHHLELAPGIERLGKIEEQFM0GPPIPLY
FG06166.1 	1IYIACVSSAHNVCPKGYWIAIVSTIAETTANHHVELQAGLERLGKIEEQFM0GPPIPVY
AN5895.1  	~IYVAMVSSAHNVCPKGYYIAIVSTIAETDSNHHLELEPGFERLGDIEEKFF~PPPIPLY
          	 **:* *****.******:********** :***:** .*:****.***:*:  ****:*

MG07137.1 	APIEDGTSSGIFISKSYDATSHFETTTD~DVKDIYRRATGEELKVEGLRD-LQVAGDE
NCU05288.1	EPLEDGRNDNIFISKSYDATSHFETSTE~DVKDIYRRCAGEELVVEGLREGIQMSQE-
FG06166.1 	EPLEDGSKDNIFISKSYDATSHFETNTD~DVKDIYRRATGEELKVEGLREGIQVAEEQ
AN5895.1  	QPLEDGKKDNIFISKSYDATSHFETTTD1DVRDIYRRATGEELVVEGLREGQTLAQE-
          	 *:*** ...***************.*: **:*****.:**** *****:.  :: :
```
